# Implementing the LifeSkills Training drug prevention program: factors related to implementation fidelity

**DOI:** 10.1186/1748-5908-3-5

**Published:** 2008-01-18

**Authors:** Sharon F Mihalic, Abigail A Fagan, Susanne Argamaso

**Affiliations:** 1 Institute of Behavioral Science, University of Colorado, Boulder, CO, USA; 2 Dept. of Criminology and Criminal Justice, University of South Carolina, Columbia, SC, USA

## Abstract

**Background:**

Widespread replication of effective prevention programs is unlikely to affect the incidence of adolescent delinquency, violent crime, and substance use until the quality of implementation of these programs by community-based organizations can be assured.

**Methods:**

This paper presents the results of a process evaluation employing qualitative and quantitative methods to assess the extent to which 432 schools in 105 sites implemented the LifeSkills Training (LST) drug prevention program with fidelity. Regression analysis was used to examine factors influencing four dimensions of fidelity: adherence, dosage, quality of delivery, and student responsiveness.

**Results:**

Although most sites faced common barriers, such as finding room in the school schedule for the program, gaining full support from key participants (i.e., site coordinators, principals, and LST teachers), ensuring teacher participation in training workshops, and classroom management difficulties, most schools involved in the project implemented LST with very high levels of fidelity. Across sites, 86% of program objectives and activities required in the three-year curriculum were delivered to students. Moreover, teachers were observed using all four recommended teaching practices, and 71% of instructors taught all the required LST lessons. Multivariate analyses found that highly rated LST program characteristics and better student behavior were significantly related to a greater proportion of material taught by teachers (adherence). Instructors who rated the LST program characteristics as ideal were more likely to teach all lessons (dosage). Student behavior and use of interactive teaching techniques (quality of delivery) were positively related. No variables were related to student participation (student responsiveness).

**Conclusion:**

Although difficult, high implementation fidelity by community-based organizations can be achieved. This study suggests some important factors that organizations should consider to ensure fidelity, such as selecting programs with features that minimize complexity while maximizing flexibility. Time constraints in the classroom should be considered when choosing a program. Student behavior also influences program delivery, so schools should train teachers in the use of classroom management skills. This project involved comprehensive program monitoring and technical assistance that likely facilitated the identification and resolution of problems and contributed to the overall high quality of implementation. Schools should recognize the importance of training and technical assistance to ensure quality program delivery.

## Background

The recent focus of school-based delinquency prevention efforts has been to identify and replicate effective research-based programs, i.e., programs that have been tested rigorously and achieved positive results in the prevention or reduction of delinquent behavior and substance use. Several programs have emerged as exemplary in meeting these criteria, and have been placed on government and private agency "what works" lists for entities seeking to implement evidence-based programs [1-6]. Once an organization chooses a model program, it expects to achieve outcomes similar to those found in research trials, contingent upon being able to implement the program with integrity to the designed model.

What is missing from this formula, and what has become increasingly more important in prevention research [7-9], is how model programs go from package to process, and how to ensure that these effective programs, once immersed in "real world" settings, are implemented as intended. Although a growing area of study, program "integrity" or "fidelity" – including adherence to critical components, methods of delivery, and program dosage – has been relatively neglected in the prevention research literature [10-13]. Particularly lacking are studies that describe how well programs are implemented, as well as what factors inhibit or promote implementation with fidelity [14-17].

### Implementation fidelity of school-based prevention programs

Schools are an ideal environment for widespread dissemination of successful delinquency prevention programs because they contain a universal target population and valuable program facilitators (i.e., teachers who are already employed by the schools who will only need training in the specific program protocols). As a result, many program developers have designed and tested prevention programs that take place in school settings, and many of these programs have demonstrated evidence of positive outcomes for students [18-20].

While schools now have more choices regarding evidence-based programs that meet their needs, successful implementation of a given program is not guaranteed. For example, the National Study of Delinquency Prevention in Schools demonstrated great variability in the implementation of school-based prevention programs, with prevention activities often not implemented with sufficient strength and fidelity to produce a measurable difference in the desired outcomes [21]. In this study, only one-half of drug prevention curricula and one-fourth of mentoring programs met dosage requirements because schools offered fewer and less frequent sessions than were specified by program developers. Moreover, only one-half of the programs were taught in accordance with the recommended methods of instruction. One national assessment of school-based prevention programming also demonstrated significant deviations in program implementation, with schools frequently operating with untrained teachers, without the required materials, and with misspecification of the population to be served (e.g., targeting high-risk students with universal programs) [22]. Only 19% of all school districts surveyed faithfully implemented effective prevention curricula.

These findings contrast with research trials that reported high rates of implementation fidelity [23-28]. For example, a program evaluation of the LifeSkills Training (LST) program demonstrated that instructors taught an average of two-thirds (68%) of the program objectives [23]. Likewise, an evaluation of the Early Alliance program demonstrated that program staff taught an average of 80% of the required material [25].

The less successful results found in community-based replications suggest that variability in fidelity increases when programs are widely disseminated [29,30]. When implementation suffers, communities are less likely to achieve the anticipated benefits of the program. While there is tension between those who promote strict adherence to program fidelity and those who promote local adaptation, our own emphasis is on maximizing fidelity. There is strong evidence that some programs *only *work when implemented with a high degree of fidelity, and other research suggests that closer adherence to core components results in stronger participant outcomes [23,28,31-36]. Proponents of adaptation have a tendency to substitute program sustainability for program effectiveness as the outcome criteria. Local adaptation may well increase the likelihood of sustaining a program, but if it renders the program ineffective, this is not a desirable outcome. Both fidelity and sustainability are necessary to an effective prevention effort [8].

### Factors promoting implementation fidelity

As programs become more widely disseminated, the need to identify factors promoting or inhibiting implementation quality becomes essential. Much of this research has been exploratory, typically based on process evaluations and qualitative evidence [37]. Nonetheless, several factors have been identified as associated with implementation fidelity, including in-depth training for program implementers, strong support from key participants, characteristics of the program itself, and comprehensive implementation monitoring.

Staff training is critical for success because it provides the knowledge and skills needed to implement the program, fosters support and commitment to the program, and communicates the importance of program fidelity [38-42]. Booster training sessions can help ensure continued program involvement, rekindle commitment where needed, and ensure that implementers are continuing to deliver the program elements with fidelity [39,43]. Studies have demonstrated a relationship between teacher training and greater implementation fidelity [38,44,45] and better student outcomes [46-48].

It is essential that program staff at all levels of implementation provide strong support for a newly chosen program. At the top level, the project director or coordinator champions the program replication from its inception and throughout implementation. Program fidelity is strongly influenced by the commitment displayed by the site coordinator, who advocates for the program, ensures that program protocols are in place, and identifies and helps resolve implementation problems [39,40,49-51]. School administrators also must back the program, and agree to adopt the initiative, make needed resources available, garner initial staff "buy-in" to the values and ideals of the program, and exert strong, continuous pressure for implementation [40,43,51]. Success or failure of school-based programs may ultimately rest with its teachers. In order to support a program that utilizes valuable class time, teachers must believe the program is worthwhile, have a sense of ownership for it, encourage implementation by others, and feel supported by school administrators [39,41,52].

Specific program characteristics also can influence the quality of implementation. Program complexity and structure have been associated with successful delivery; programs with clear goals and procedures are easier to implement and less likely to result in deviation [40,49,52,53]. A set curriculum with activities that are viewed as relevant, attractive, and easy to use also enhances program adoption, helps provide a clear program structure, and may reduce deviations from the intended content [42,48]. Integration into the school system, particularly finding a regular class for programming, is important for adoption, implementation, and sustainability [40].

Finally, ongoing and rigorous program oversight is associated with implementation fidelity [25,28,32,54,55]. An evaluation of the Early Alliance program attributed high levels of implementation adherence to program monitoring protocols, which included intensive staff training, implementers' self-reports of content taught each session, weekly staff supervision, and other technical assistance from research staff [25]. In contrast, an evaluation of the Multisystemic Therapy (MST) model indicated more program drift and greater therapist variability when standard weekly feedback from MST consultants was eliminated [32]. Likewise, an attempt to disseminate the LST program in Kentucky reported that only one-half of teachers who received training later taught lessons, which the authors attributed to a lack of oversight by state and local school administrators [56].

In summary, prior literature has described mixed evidence regarding the extent of implementation fidelity of school-based prevention curricula, with some research trials documenting high levels of implementation fidelity, and community-based replications typically achieving far less success. Though some factors related to implementation quality have been identified, very little is known regarding how program activities actually take place during replications, what specific challenges are faced, and how these problems can be overcome [57]. These are all relevant issues for communities interested in replicating evidence-based programs, and more information can help guide future efforts and increase the likelihood that communities will satisfy program requirements.

The Blueprints Initiative, funded by the Office of Juvenile Justice and Delinquency Prevention, U.S. Department of Justice, was designed to accomplish these goals [2]. Blueprints model programs have been held to the highest standard of scientific testing and controlled program replication, and the Blueprints Initiative examined how these programs were replicated in multiple, naturalistic settings. Earlier findings identified factors likely to relate to implementation fidelity, including program support and commitment among administrative and implementing staff, training and technical assistance, specific elements of the program itself, and characteristics of the adopting organization [54,58].

The current paper expands upon earlier published findings regarding the process evaluation of one model program, the LST school-based drug prevention curriculum [54]. The previous results were based upon replication of LST in 70 sites (292 schools) across the United States. Primarily descriptive data were analyzed in order to determine the extent to which schools replicated the LST curriculum with strong adherence to the model, identify problems faced during implementation, and describe the steps taken to overcome these challenges. After two years of implementation, teachers were observed to have taught 81–86% of the required LST objectives and activities. Implementation factors that were significantly correlated with higher rates of implementation fidelity included the support and ability of the local coordinator and observations that teachers spent much time using didactic instruction (though this measure was also correlated with worse student behavior and less student participation in lessons). Variables significantly related to teaching all the lessons (i.e., program dosage) included teachers' overall rating of the program and quality of the materials.

The current paper summarizes results from the complete LST replication project. We describe implementation outcomes for the full sample of 105 sites (432 schools) after replication of the entire three-year curriculum in all sites. In addition to providing a descriptive analysis of implementation fidelity results (including challenges faced and overcome), we use multivariate analysis to demonstrate predictors of four primary elements of implementation fidelity (adherence, dosage, quality of delivery, and participant responsiveness). Four research questions are addressed:

1) Did the LST program reach the intended, universal population of middle school students?

2) To what extent was the program implemented with fidelity; i.e., covering the majority of information and activities in each lesson, delivering all the lessons, using varied teaching techniques, and engaging participants?

3) What factors were associated with these four aspects of implementation fidelity?

4) What obstacles and barriers were encountered during implementation, and how were they addressed?

## Methods

### The LifeSkills Training initiative

The LST process evaluation was conducted by Blueprints project staff at the Center for the Study and Prevention of Violence (CSPV), located at the University of Colorado. CSPV's primary goal is integrate prevention research and practice. The "hallmark" project of CSPV has been the Blueprints for Violence Prevention Initiative, an effort to identify and promote the implementation of exemplary evidence-based programs. National Health Promotion Associates (NHPA), Inc, the providers of the LST curriculum, and their cadre of certified LST trainers, were contracted to provide training and technical assistance to implementation sites. Site selection occurred from 1999 to 2001, with the final sample including 105 sites and 432 schools. Sites were comprised of one to 24 schools, and sometimes included multiple school districts. Sites were located in urban, suburban, and rural areas and served students of varying socioeconomic status and racial/ethnic backgrounds. (See Additional File 1 for more information regarding sites and schools participating in the project.)

The LST program is a school-based, universal program designed to prevent tobacco, alcohol, and other drug use among middle and junior high school students. Research trials have demonstrated that the program reduces tobacco, alcohol, and marijuana use up to 80%, with effects sustained through high school and demonstrated for adolescents of varying socioeconomic status and race/ethnicity [33]. The three-year program includes self-management skills (e.g., decision-making, coping with anxiety), social skills (e.g., communication, assertiveness), and information relating to drug use (e.g., consequences of drug use, drug resistance skills). Lessons are generally taught by classroom teachers using a variety of teaching techniques, including didactic instruction, classroom discussion, behavior skill rehearsals, and demonstration of skills.

Schools participating in the Blueprints Initiative did not receive monetary incentives to replicate LST, but were provided with all curriculum materials, training and technical assistance needed to implement the curriculum. Thus, participating schools were able to provide LST to all eligible students with no direct costs (other than staffing) to the school district. In exchange, schools were required to implement the full three-year curriculum. The first year (level one) included 15 lessons to be taught to all sixth- or seventh-grade students, one to five times per week in at least 50-minute class periods. In the second year of implementation, these students were to receive ten booster sessions (level two), while an incoming cohort of sixth- or seventh-grade students would receive the level one curriculum. In the third year of implementation, eighth- or ninth-grade students received five booster sessions (level three), seventh- or eighth-grade students received the level two curriculum, and an incoming cohort of sixth- or seventh-grade students received the level one curriculum. During the research project, violence prevention lessons (three lessons in level one, two in level two, and four in level three) were added to the packaged curriculum. As NHPA considered these lessons optional, and schools had not previously committed to teaching them, the lessons were not required from Blueprints sites.

### Site Selection

Sites responded to a Request for Proposal (RFP) issued by the Office of Juvenile Justice and Delinquency Prevention and/or applied directly to CSPV (Blueprints). Applications provided program implementation details, including the subject in which LST was to be taught, class size, names of instructors, timelines, and other site-specific information. Each site was asked to identify a local coordinator to monitor program activities, help overcome challenges, and communicate with CSPV (Blueprints) and NHPA. Written letters of commitment from school principals and superintendents also were required. (See Additional File 1 for more information describing the site selection process.)

Feasibility visits were then conducted by CSPV (Blueprints) staff and certified LST trainers from NHPA to verify application information, describe the core elements of the program, explain the research requirements of the project (with a strong emphasis on the need to implement the program with fidelity), assess commitment to implement LST with fidelity, and address local concerns. Selection decisions were based on site readiness and ability to replicate the program. Given the small number of applications received, most sites were accepted into the study, but those that were clearly unprepared (e.g., demonstrating little support from administrators and/or teachers) or were unable to fulfil the project's requirements (e.g., unable to allow observations of lessons) were not selected.

### Teacher training workshops

Each site received a two-day training workshop in the first year of implementation, and a one- or two-day workshop in the second and third years to familiarize staff with the program rationale and the key components of each lesson. Training was required for all LST instructors and local coordinators, and was encouraged for school administrators and other support staff. (See Additional File 1 for more details regarding LST training workshops provided in the Blueprints Initiative.)

### Technical assistance (TA)

Technical assistance with program issues was provided by LST trainers from NHPA. As part of the process evaluation, CSPV (Blueprints) staff visited sites once per year to conduct informal interviews with LST program coordinators, principals, classroom observers, and some teachers. Discussions focused on the progress of implementation, including support for the curriculum, problems encountered, and solutions achieved. Staff also observed LST classes, usually in conjunction with local observers, to assess the reliability of their information. CSPV (Blueprints) and NHPA staff provided telephone-based technical assistance (TA) to local coordinators as needed during the school year, focusing on implementation progress and achieving solutions to implementation challenges. At the end of each school year, CSPV (Blueprints) provided each site with a written report describing the overall project results, as well as site-specific information regarding the extent of implementation fidelity achieved, obstacles faced and overcome, and recommendations for improvement. Schools could request phone, email, or on-site TA from NHPA trainers throughout the project. (See Additional File 1 for more detail regarding the provision of TA.)

### Measures

The independent variables included in the analyses were largely derived from prior research that assessed implementation fidelity of eight Blueprints programs (not including LST), replicated in 42 sites [58]. Variables in this study include ratings of the program training workshops, characteristics of the LST program, school-level characteristics, administrative support, staff buy-in, parent awareness of the program, quality of the local coordinator, time spent teaching classes, and student behavior. Most independent variables were based on self-reports from LST instructors or site coordinators, though one measure each was obtained from LST trainers, CSPV staff, and local classroom observers. Variables were coded so that higher scores reflected more successful implementation fidelity. Descriptive statistics for all variables are given in Table 1, and individual measures are described in more detail below.

**Table 1:** Independent variables and their association (r) with dependent variables

**Variable^1^**	**No. of Items**	**Range**	**SD**	**Mean Score**	**r – Implem. Score**	**r – Teach All**	**r – Interactive**	**r – Student Particip**.
**LST Training Quality**	3	2.80–4.97	0.39	4.31	-0.14	-0.14	-0.01	-.07
**LST Program Characteristics**	4	1.80–5.00	0.71	3.42	0.14	0.20*	-0.05	.09
**Program Coordinator**	1	1.00–3.00	0.67	2.16	0.16	-0.02	0.12	.11
**School Characteristics**	13	1.92–5.00	0.65	3.89	0.05	0.03	0.10	.07
**Admin. Support**	1	2.89–5.00	0.48	4.22	0.11	0.09	-0.13	.24*
**Teacher Support**	1	2.74–5.00	0.47	3.68	0.14	0.01	-0.20*	.17
**Parental Awareness**	1	1.00–4.67	0.62	3.05	0.20*	-0.07	0.06	.32**
**Length of Class (minutes)**	1	32.5–68.1	5.42	48.22	0.15	0.06	0.12	.03
**Student Behavior**	1	2.78–4.82	0.37	4.02	0.55**	-0.04	0.28**	.19

**^1 ^***All variables are coded so that higher scores represent better outcomes*.

* Pearson Correlation is significant at the .05 level (2-tailed).

** Pearson Correlation is significant at the .01 level (2-tailed)

Teacher reports were based on written mail surveys conducted at the end of each program year, which were collected and sent to CSPV by site coordinators. All surveys were conducted anonymously, and response rates were fairly high: over the three years, about 70% of teachers completed year-end surveys. Multiple teachers implemented LST during the three-year study, though some teachers participated each year and may have responded more than once. Teacher reports were averaged to create site-level scores for each implementation measure. Both independent and dependent variables were assessed at the site level, rather than for individual teachers. This procedure was used because the study aim was to examine the ability of schools as a whole to replicate the LST program with fidelity, and certain site-level characteristics were expected to influence implementation procedures. Scores also were collapsed across program years because each year of implementation covered similar themes and topics. Additionally, feedback on implementation was provided in annual reports to all sites, and all information in these reports was collapsed at the site level to avoid embarrassment to individual teachers in small schools, as well as any repercussions that might occur at the administrative level due to inadequate or incomplete implementation by a teacher.

Written mail surveys were completed by local site coordinators at the end of the three-year project. Coordinators reported on 42 items related to program implementation, characteristics of the local school district and program implementers, training and technical assistance, and support for the program. Each item was rated on a five-point scale identifying the extent to which it was a "significant barrier" (rating of "one") or "significant asset" (rating of "five") to implementation as a whole, throughout the project. In all, 104 of the 105 surveys were completed by local coordinators.

### Training quality

The overall quality of the training workshop was measured from reports by the site coordinators at the end of the three-year period, teacher reports conducted at the end of each training workshop, and trainer surveys also collected at the end of the workshop. Coordinators rated the overall quality of training workshops from one ("significant barrier to implementation") to five ("significant asset to implementation"). Teachers and trainers rated the workshop on a five-point scale (from "poor" to "excellent"). The three reports were averaged to form the training quality measure (Cronbach's alpha of 0.49).

### LST program characteristics

Coordinators rated the extent to which four characteristics related to the LST program (the quality of the materials, flexibility, time required, and complexity) were a barrier (score of one) or asset (score of five) to implementation. These items were combined to form the program characteristics scale (alpha of 0.70).

### School characteristics

The school characteristics scale (alpha of 0.87) was derived from 13 items rated by coordinators, including staff participation, administrative support, open communication between agency staff, fit between program and agency, cohesion and collaboration, clarity of goals, clear lines of authority, structural stability, champion, facilities, financial support, resources for program, and political climate. Each item was rated on a five-point scale (from "significant barrier" to "significant asset" to implementation).

A separate measure was derived from teachers who reported the degree of administrative support for LST, rated on a five-point scale, from "not at all supportive" to "very supportive."

### Teacher support

Teacher support was based on instructors' overall rating of the LST program on a five-point scale, from "poor" to "excellent."

### Parent awareness

Teachers reported the degree to which parents were aware of the program on a five-point scale, from "unaware" to "very aware."

### Program coordinator

The overall quality of the LST coordinator was rated by Blueprints staff using a three-point scale ("poor", "average", and "excellent").

### Length of LST class

Teachers reported the average length of their LST classes in minutes.

### Student behavior

Classroom observers rated student behavior during lessons on a five-point scale, from "poor" to "excellent."

### Dependent variables

Prior studies have identified four primary elements of implementation fidelity: adherence, dosage, quality of delivery, and participant responsiveness [10]. In this project, we created a measure for each of these four domains of fidelity. Classroom observations of teachers' delivery of the LST curriculum measured adherence to the curriculum ("implementation score"). CSPV contracted with one or two local consultants at each site to assess implementation fidelity through classroom observations of lessons. To avoid bias, observers were not school staff. The only qualifications required were an interest in youth prevention and having the available time to devote to the project. The observers attended LST training workshops to meet instructors and learn about the curriculum. Written instructions were provided for completing the LST fidelity checklist, and CSPV representatives conducted telephone conversations with observers after training to ensure that they were prepared to begin classroom observations. The observers then were asked to attend four (26%) of the 15 classroom sessions taught by each LST instructor during level one, three (30%) level two lessons, and two (40%) level three sessions. During each observation, the proportion of objectives and activities taught was identified using a fidelity checklist designed by the program developer and used in prior evaluation trials and program replications of LST [14,23,28,33,56] An implementation score for each lesson taught was calculated as the percentage of material taught out of all required material. For example, a lesson in which five of ten required objectives were delivered received an implementation score of 50%. Average implementation scores were then created for each site, based on all teachers and years of implementation observed for that site. Implementation scores for two sites that withdrew prior to year-one implementation could not be calculated. Observers also were asked to identify the use of varied instructional techniques, assess student participation, and note any problems, such as deviations from the curriculum, student behavior issues, or inadequate facilities. Observations were not scheduled in advance with teachers, and observers were instructed to refrain from participating in the lesson or interacting with students to preserve the naturalistic classroom setting. Blueprints staff supervised observers by reviewing observation procedures in phone calls and written correspondence prior to implementation, talking to observers during implementation about their work, and conducting joint observations annually.

During yearly site visits, Blueprints staff conducted classroom observations with the local observers to validate the accuracy of the information. During the three-year project, 302 joint observations were conducted. Ratings were compared on each pair of implementation checklists. The observer and staff correspondence across all levels and years of implementation was 89.7%, indicating a high level of reliability of the observer information.

LST dosage ("teach all") was based on a question in the year-end teacher surveys that asked instructors to check all lessons that they taught during the year. This question was then coded as a dichotomous measure. If a teacher had taught every lesson, s/he received a score of one; if not, a score of zero was given. An average score was created for each site, based on all teachers and years of implementation.

Quality of delivery ("interactive") was assessed as the percentage of the class period spent using the three recommended interactive teaching techniques (classroom discussion, skill demonstration, and behavioral rehearsal). This measure was reported by classroom observers on the fidelity checklists. A summary score was created for each site, based on all site observations over the three-year period of implementation.

Participant responsiveness ("student participation") was measured by teacher year-end survey responses to the item: "What percent of students participated in LST activities that you taught?" A summary measure was created for each site, based upon the responses from all LST instructors at the site and averaged across the three years of implementation.

### Data analysis

Results for the research questions are based on teacher and coordinator surveys, observations of lessons (from consultants and Blueprints staff) and qualitative interviews conducted by research staff with key participants. Results are primarily descriptive in nature. The third research question, identifying predictors of implementation fidelity, was analyzed using quantitative data from written surveys and observations. Multiple linear regression was used to identify predictors of the four elements of implementation fidelity. All independent variables were entered into the model simultaneously, and significant predictors (p < 0.05) were identified.

## Results

### Did the LST program reach the intended, universal population of middle school students?

A prerequisite of site selection was that schools implement the program with *all *eligible students. At the beginning of each school year, schools were required to submit schedules of implementation that identified the dates and times during which LST would be offered to the targeted population (all 6th–8th or 7th–9th grade students). Schedule adherence was verified by the local classroom observers at each site. When problems arose that prevented teachers from reaching all students, Blueprints staff were usually notified by the local observer, and efforts would be made to resolve the problems. Typically, the lack of instruction was due to a lack of trained teachers (caused by staff turnover after the initial training). In these cases, a second training or TA visit was held to train additional instructors. Sometimes, schools or teachers delayed in teaching students due to scheduling problems or lack of enthusiasm. For these cases, Blueprints staff worked with local coordinators to motivate instructors to begin teaching the program.

Although 100% exposure was not obtained in every school, all efforts were made by Blueprints staff to ensure the program was delivered to the intended population and that all eligible students received the program. For the most part, this was accomplished successfully. During the three years of implementation, the LST curriculum reached approximately 172,355 students.

### To what extent was the LST curriculum implemented with fidelity?

Our primary measure of implementation fidelity was teachers' adherence to the LST curriculum, defined as the proportion of critical objectives and activities taught during observed lessons. As shown in Table 2, instructors were observed to closely follow the curriculum. The average site adherence score of 86% indicates that 86% of the required material was taught by teachers in participating schools during the three-year project. High rates of curriculum adherence were demonstrated for all three levels of the LST program, with average fidelity scores of 86% for the level one, 85% for level two, and 88% for level three. Deviation in adherence across sites was not great, as overall scores ranged from 64%–98%. However, individual teachers varied in the extent to which they taught the critical program objectives. Individual teachers were observed to teach between 0%–100% of the required information (results not shown).

**Table 2:** Implementation fidelity results

**Outcome**	**Mean**	**Range**
**Overall Adherence Score^1^**	86%	64–98%
***Level 1***	86	57–100
***Level 2***	85	33–100
***Level 3***	88	64–100
**Taught All Lessons^2^**	71%	
***Level 1***	77	-
***Level 2***	75	
***Level 3***	60	
**Use of Teaching Techniques^1^**		
***Discussion***	37%	23–58%
***Lecture***	32	13–58
***Behavioral Rehearsal***	20	8–33
***Demonstration***	12	0–28
**Student Participation^2^**	89%	63–100%
***Level 1***	92	15–100
***Level 2***	88	28–100
***Level 3***	85	25–100

^1^Reported by classroom observers

^2^Reported by classroom instructors

Program dosage – whether or not all lessons were taught, and the average length of lessons – was reported by teachers in year-end surveys. As shown in Table 2, 71% of teachers reported teaching all required LST lessons (15 in level one, ten in level two, and five in level three). This outcome varied by level (year) of implementation, with 77% of level one LST instructors completing all level one lessons, compared to 75% of level two teachers, and 60% of level three teachers. Although we cannot state with any certainty why the drop occurred in year three, we did receive reports from many teachers that the booster lessons were repetitive with information in prior years. Teachers reported an average class length of 48 minutes (with a range of 33 to 68 minutes, as shown in Table 1), which closely matched the dosage requirement that LST lessons be a minimum of 50 minutes.

A key aspect of the LST curriculum is variation in instructors' teaching techniques that includes didactic instruction, discussion, demonstration, and behavioral rehearsal as appropriate during lessons. According to observer reports, teachers spent, on average, 37% of class periods facilitating student discussion, 32% using didactic instruction, 20% conducting behavioral rehearsals, and 12% demonstrating skills. Teachers reported high participant responsiveness to the program. On average, across the sites, 89% of the students participated in lessons.

### What factors were associated with implementation fidelity?

As shown in Table 1, teachers and coordinators reported high ratings of the independent variables hypothesized to relate to the quality of implementation fidelity in this project. As rated by teachers, coordinators, and LST trainers, the quality of the training workshops was "good" (4.31 on a five-point scale). Similarly high ratings were given for the LST program overall (rated by site coordinators as 3.42), support for the program from both school administrators (4.22) and teachers (3.68), and healthy school environments (3.89).

These variables demonstrated modest bivariate correlations with the dependent variables that measured implementation fidelity of the LST curriculum (see Table 1). Higher implementation scores were associated with higher ratings on all independent variables except the quality of the training workshop (r = -0.14). Of these measures, parental awareness of the program and student behavior were significantly related to the implementation score. Characteristics of the LST program were significantly related to dosage ("teach all") because teachers were more likely to teach all the lessons if the curriculum was of high quality, flexible, and easy to use (as rated by coordinators). The use of interactive teaching techniques was significantly associated with better student behavior, but less teacher support of the program. Student participation was statistically correlated with greater parental awareness of the LST program and strong administrative support.

Tables 3, 4 and 5 present the results of the multivariate analyses used to assess the relationship between the independent variables and the four measures of fidelity: adherence, dosage, quality of implementation delivery and participant responsiveness. As shown in Table 3, two of the nine independent variables were significantly (p < 0.05) related to the implementation adherence score, with the quality of the LST program and better student behavior related to a greater proportion of material taught by teachers. Two variables were marginally related (p < 0.10) to adherence. Longer LST classes and the quality of the LST coordinator were associated with greater fidelity to the curriculum. Several variables were not significantly related to the adherence score, including training quality, characteristics of the school environment, administrator support, teacher support, and parental awareness of the program.

**Table 3:** Factors related to implementation adherence – implementation score

**Independent Variable**	**B**	**SE**	**Beta**
**LST Training Quality**	-2.38	1.53	-0.14
**LST Program Characteristics**	1.89	0.89	0.19**
**Program Coordinator**	1.47	0.88	0.14*
**School Characteristics**	-0.77	0.99	-0.07
**Administrative Support**	1.26	1.39	0.09
**Teacher Support**	1.30	1.37	0.09
**Parental Awareness**	-0.86	1.08	-0.80
**Length of Class**	0.20	0.11	0.16*
**Student Behavior**	9.96	1.62	0.53**
			
***Constant***	32.48**	12.24	-
***R-squared (Adjusted)***		0.40 (0.34)	

*All variables are coded so that higher scores represent better outcomes*.

* p < 0.10 **p < 0.05

**Table 4:** Factors related to implementation dosage – teach all lessons

**Independent Variable**	**B**	**SE**	**Beta**
**LST Training Quality**	-0.09	0.07	-0.15
**LST Program Characteristics**	0.08	0.04	0.22**
**Program Coordinator**	0.01	0.04	0.02
**School Characteristics**	-0.02	0.04	-0.04
**Administrative Support**	0.08	0.06	0.16
**Teacher Support**	-0.01	0.06	-0.02
**Parental Awareness**	-0.06	0.05	-0.16
**Length of Class**	0.00	0.00	0.09
**Student Behavior**	-0.00	0.07	-0.01
			
***Constant***	0.60	0.52	-
***R-squared (Adjusted)***		0.09 (0.002)	

*All variables are coded so that higher scores represent better outcomes*.

* p < 0.10 **p < 0.05

**Table 5:** Factors related to implementation quality of delivery – interactive teaching

**Independent Variable**	**B**	**SE**	**Beta**
**LST Training Quality**	0.39	0.80	0.05
**LST Program Characteristics**	-0.13	0.47	-0.03
**Program Coordinator**	0.29	0.46	0.06
**School Characteristics**	0.56	0.53	0.12
**Administrative Support**	-0.58	0.73	-0.09
**Teacher Support**	-1.47	0.72	-0.23**
**Parental Awareness**	0.28	0.57	0.06
**Length of Class**	0.05	0.06	0.09
**Student Behavior**	2.38	0.85	0.28**
			
***Constant***	32.48**	12.24	-
***R-squared (Adjusted)***		0.17 (0.09)	

*All variables are coded so that higher scores represent better outcomes*.

* p < 0.10 **p < 0.05

Table 4 shows the relationship between independent variables and implementation dosage (i.e., teaching all required lessons). The quality of the LST program was the only variable significantly related to dosage, and is an indication that coordinators' positive views of the program were associated with teaching all required lessons.

The results in Table 5 demonstrate a significant relationship between better behaved students and teachers' greater use of interactive methods. Since data are cross-sectional, however, it cannot be determined whether using interactive teaching techniques led to better student behavior, or whether better student behavior was conducive to greater use of these techniques. Less intuitively, teachers who were more supportive of the LST program were less likely to use interactive teaching techniques.

None of the independent variables were statistically related to the last measure of implementation fidelity, student participation. Results are not presented.

### What obstacles and barriers were encountered during implementation, and how were they addressed?

The quantitative ratings cannot capture the depth or range of experiences faced by schools and instructors when implementing the curriculum. The next section identifies the general and specific challenges that were faced during the project, describes how school personnel responded to them, and assesses the extent to which challenges were overcome during the three years of LST implementation. Information is largely based on the qualitative data obtained during site visits by Blueprints and NHPA representatives.

### Implementation failures

Implementation failures occurred throughout the three years of the project, when sites or schools were unable to successfully implement the LST curriculum or fulfill the research requirements. Full implementation failure occurred in six sites, representing seven schools. One site withdrew prior to year-one training because of a major reorganization in the school district that temporarily closed the charter school where LST was to be implemented. Another site began implementation but withdrew during year one, and the other four sites withdrew from the project during year two, usually before receiving an LST booster training.

Of the six site failures, two occurred at sites in which outside prevention agencies had applied for the grant and were delivering the program. Funding problems within these agencies and miscommunication between the school and the agency were related to failure, as was lack of strong principal support. The other four failures were related to either administrative changes and lack of buy-in from new principals or problems with integrating LST into the school schedule.

In addition, 22 schools from 17 other sites withdrew from the project over the three years of implementation (nine of these schools withdrew prior to year one training and implementation). These cases often were related to low or no teacher attendance at required LST training workshops. As explained below, this challenge was faced to some degree by many schools; however these failures represented an extreme problem, or multiple problems, that could not be resolved. Every effort was made to provide support to schools and sites that considered withdrawing from the project, but TA did not always help these sites. For example, make-up staff training workshops were held, but in sites facing organizational upheavals or communication failures, second trainings often were no more successful in ensuring teacher attendance than the initial training workshop.

### Teacher training workshops

Although all LST instructors were required to attend training workshops, absenteeism often occurred. When absences signaled a clear lack of commitment from the site (e.g., if all teachers from a school were missing), schools were asked to withdraw from the project. If absenteeism reflected a lack of communication between school personnel, such as administrators failing to provide substitute teachers, or scheduling other required workshops on the same day, sites were offered make-up trainings. Staff turnover after training was common, and typically delayed implementation until another training opportunity could be arranged. In a few cases, sites did not identify the teacher turnover, and either allowed untrained instructors to deliver the lessons, or chose not to deliver the program to the teacher(s)' classes. Schools could avoid implementation delays by sending additional staff (particularly guidance counselors) to trainings who could teach lessons if needed, but doing so was often difficult for schools to arrange.

### Integrating the LST curriculum into the school schedule

Many schools struggled to integrate the three-year LST curriculum in their existing schedules, particularly as the program was to be received by all students, ideally in classes lasting at least 45 minutes with fewer than 35 students. The most common barrier to integration was finding time outside of "core" academic subjects, and this challenge increased during the project, just as academic pressures to fulfil standardized test requirements also increased. Placing LST in non-academic subjects was not always the best solution. When students realized their elective courses would be used to teach a curriculum, they could be critical of the program and disruptive during lessons, which was significantly associated with lower teacher adherence to the curriculum. Some schools scheduled LST into short homeroom periods or other free periods, which resulted in less time to teach required material (also negatively associated with implementation fidelity) and often to student behavior problems.

Implementation during physical education classes was a common, but not effective solution to scheduling difficulties. Classes were sometimes combined during physical education (PE) and resulted in class sizes of nearly 100 students in one site, which could exacerbate behavior problems. Moreover, participation in discussions and role plays suffered in large classes, as not all students could participate, and some were reluctant to share personal experiences in front of large groups of their peers, particularly those they did not already know. Finally, some PE classes were held in the gym, cafeteria, or even outside, and were a distraction for both students and teachers.

Over the three years of the project, many sites integrated the program into their curriculum, sometimes by trying various arrangements until finding a niche. Particularly successful strategies included identifying a subject, such as health, in which similar information was being taught, and replacing that material with LST lessons, or matching the LST curriculum with state or district teaching requirements so that all school personnel felt the time was well spent and not viewed as additional class work.

### Student misbehavior

Although teachers and observers generally reported good student participation in lessons, student behavior problems nonetheless occurred with frequency. These issues were especially apparent in large classes and during discussions and behavioral rehearsals. In turn, misbehavior led some teachers to avoid interactive exercises – or to spend too much time managing student behavior problems, which resulted in less time to cover the required material.

Classroom behavior problems were difficult to overcome, particularly for instructors from outside the school system who were unfamiliar with students. In these cases, classroom teachers were asked to help manage student behavior. Likewise, if possible in large, combined classes, teachers divided the workload so that one teacher taught while the other monitored behavior. In extreme cases, sites (or research staff) requested TA from LST trainers, who modeled teaching techniques designed to *prevent *student misbehavior, and/or reviewed strategies for facilitating discussions and behavioral rehearsals while still covering required material.

### Lack of support for the program

While the measures of support from coordinators, school administrators, and teachers were not strongly related to program fidelity in multivariate analyses, support from key participants could influence program monitoring, training workshops, and other implementation procedures. Enthusiastic local coordinators were able to provide the onsite monitoring and proactive problem-solving that external research staff could not. Conversely, coordinators who lacked interest in the program typically failed to monitor program activities and intervene when needed, such as identifying teacher turnover and arranging new training workshops, or ensuring that LST schedules were being followed. Other coordinators lacked authority to effectively manage the program. When classroom teachers or guidance staff acted as coordinators, ensuring full teacher attendance at training workshops or scheduling LST classes could be difficult, as this required approval from school administrators. Coordinators who were too far removed from the classroom (e.g., those in school district offices) were often not perceived as credible by teachers, and thus had difficulty communicating with instructors or offering assistance.

School principals and district administrators needed to promote the program, when adopting and integrating it in the school schedule as well as during implementation, to bolster enthusiasm from other staff and ensure that lessons were taught. Active administrators introduced the program to teachers and elicited their support, attended teacher training workshops, observed lessons, kept informed of implementation progress, and even taught classes in some cases. In contrast, other principals did not make the curriculum a priority, perhaps due to competing demands and increased pressure to raise students' academic performance. In more extreme cases, a lack of principal support could lead to site failures. In two failed sites, outside prevention agencies had coordinated the project and provided LST instructors, but had not engendered full support from administrators. As a result, when they were unable to continue teaching the program, principals refused to take on the burden. One site could not find a suitable subject in which to teach the curriculum, and the principal was unwilling to make room in the school schedule. Another failed site involved principal turnover, with the new principal overwhelmed with new duties of the job and unwilling to spend time trying to integrate the program into the school curriculum. Research staff solicited administrator enthusiasm and support by requiring principals to sign letters of commitment as part of the application process, as well as through personal visits to discuss the goals of the project, progress of implementation, and administrator involvement in the initiative.

Teacher support for the program varied by site and over time. While the majority of instructors had very positive views of the program, others resented the mandate to teach LST, particularly when their input was not solicited, and when they were overburdened with other responsibilities. Some teachers did not support LST because they felt similar material was already being taught in the school, they disliked the content or theory of the program, or they felt other drug prevention curricula were better. Other reasons for a lack of teacher support included concerns about being observed, or feeling that project guidelines regarding fidelity were too rigid and did not allow for teacher creativity and flexibility.

Teacher dissatisfaction sometimes resulted in instructors deviating from the curriculum by supplementing lessons (i.e., adding videos, "scare tactics," or other activities) or deleting information, activities, or entire lessons. Some teachers were observed telling students that they had to "get through" LST lessons before they could begin other work. Not surprisingly, students in these classrooms tended to be uninvolved and disruptive. When instructors from outside the school taught lessons, classroom teachers often appeared uninterested in the material (some were even observed reading newspapers and paying bills), and students typically responded with boredom and restlessness. Instructors' lack of buy-in also contributed to site failures. If both instructors and administrators were reluctant to champion the program, the likelihood of overcoming challenges was diminished.

During implementation, CSPV staff met with as many instructors as possible to listen to their concerns, thank them for their support, and recommend that those with problems seek technical assistance from trainers. In site visits and year-end reports, coordinators and school administrators were encouraged to foster teacher support by scheduling regular meetings with staff, and providing guidance counselors or others to co-teach lessons if teachers requested such assistance. Even if this advice was not followed, teachers who did not enthusiastically endorse the program were sometimes observed to effectively deliver lessons, which may help to explain the lack of significance between teacher support and implementation fidelity.

## Discussion

Widespread replication of effective prevention programs is unlikely to affect the incidence of adolescent delinquency, violent crime, and substance use until the quality of implementation of these programs by community-based organizations can be assured. This paper presents the results of a process evaluation focused on identifying the extent to which schools participating in the Blueprints for Violence Prevention Initiative were able to successfully implement the LST drug prevention program. In addition, the project identified factors that promoted implementation quality, challenges that were faced during replications, and the degree to which problems were overcome.

The process evaluation demonstrated very high rates of implementation fidelity among the sites and schools participating in the project. According to observer reports, sites delivered, on average, 86% of the program objectives and activities required in the three-year curriculum. Teachers were observed using all four recommended teaching practices (didactic instruction, discussion, demonstration, and behavioral rehearsal) and student participation in lessons was good. Teachers and site coordinators also reported strong implementation outcomes and satisfaction with the program. For example, end-of-year surveys demonstrated that 71% of the teachers delivered all the required LST lessons over the three-year period, class length was in accordance to dosage requirements, and satisfaction with the curriculum was better than average. Program coordinators rated the LST program favorably with regard to the quality of the materials, flexibility, time required, and complexity. Another measure of the success of the program was shown by the high rate of student participation. On average, 89% of students participated in lessons.

Factors related to each of the four components of implementation fidelity (i.e., adherence, dosage, quality of program delivery, and student participation) were also assessed in four multiple regression models. LST program characteristics and student behavior were associated with outcomes in two of the four models. Sites whose coordinators had rated the LST program favorably with regard to quality, flexibility, complexity, and time required had higher levels of teacher adherence and dosage. Program developers are increasingly producing detailed manuals that specify the nature of the required program components [57]. Attractive packaging of a curriculum and ease of use are important considerations in school-based programs. Teachers often lack the time to develop lessons and activities around violence and drug prevention. Programs that are well-developed and contain specific instructions and activities for implementation can be extremely beneficial to teachers who are already overburdened. In an earlier Blueprints project in which eight other Blueprints programs were replicated, ideal program characteristics were found to be related to higher dosage and sustainability of the program [58].

Better student behavior was related to higher teacher adherence and use of interactive teaching techniques. Teachers who spend excessive amounts of time reprimanding and striving to maintain control of a class have less time to teach the lessons and engage students in meaningful discussion and behavioral rehearsal. Teachers who are not adept at managing their classes may avoid the use of interactive techniques for fear of losing control of the class. Our own observations in classrooms showed that teachers with poor classroom management skills often lost control of the class when using interactive techniques. This suggests that schools should consider teacher training in classroom management skills prior to adopting programs that use interactive techniques, such as LST.

Two other variables were marginally related to teacher adherence: the quality of the local coordinator and a greater amount of time spent teaching each lesson. In the absence of a strong and proactive coordinator, programs can drift. A good coordinator can provide direction, leadership, and motivation to keep implementation on track. Good coordinators maintained contact with teachers, identified potential problems, and worked to resolve them before they became major obstacles. Allowing adequate time to teach each lesson was also important. Qualitative interviews with program staff provided additional support for this finding. Instructors frequently reported to research staff that it was difficult to cover all the required information, even in a 50-minute class period. Time constraints were exacerbated when teachers had to use instruction time to manage student misbehavior.

It is difficult to interpret the finding that greater teacher support for the program was related to less frequent use of interactive teaching techniques. We suspect that many of the teachers lacked the skills and experience to use the interactive methods. They may have been very motivated and supportive of the program, but unable to adjust to the more frequent use of these teaching techniques. Some motivated teachers may have limited their use of the interactive techniques in classrooms that contained students who were difficult to manage. In fact, good student behavior and the use of interactive techniques were positively correlated. LST training workshops exposed instructors to these teaching techniques; however, instruction in effective classroom management techniques was not included, and more in-depth training in the efficient use of these techniques may be necessary.

Though research on factors related to implementation fidelity is growing, this is a relatively new area of study and few other studies to date have relied on quantitative analysis to identify these factors. In the Blueprints Initiative process evaluation of 42 sites replicating eight model programs, multivariate analyses showed several factors related to implementation fidelity, including quality of technical assistance, ideal program characteristics, consistent staffing, and community support [58]. The National Study of Delinquency Prevention in Schools demonstrated significant correlations between a number of school- and teacher-level factors and program quality, including the quality of the program coordinator, integration into the school schedule, organizational support, and standardization of program materials [21].

Unlike the National Study of Delinquency Prevention in Schools, the Blueprints Initiative provided schools with program materials, teacher training, and technical assistance to replicate the LST program. In addition, sites were screened in advance to determine their readiness and support to implement the program with fidelity. Throughout the project, Blueprints staff provided program monitoring to ensure that curriculum activities were taking place and to encourage fidelity. One-third to one-fourth of all lessons were observed. Given this level of support and encouragement to implement with fidelity, implementation of the curriculum did not occur under typical, "real world" conditions. Thus, schools participating in this project may not be representative of all schools that might choose to implement LST, and the support provided may have resulted in higher implementation levels than might otherwise be observed. In fact, the results demonstrated implementation fidelity rates even higher than those found in LST research trials and some replication efforts. It may be that the most important predictor of implementation quality was the technical assistance provided, and that this support overshadowed other factors that might be more important in community-based replications that do not receive this level of technical assistance.

Given the nature of this project, the results of the multivariate analyses should be viewed with some caution. Most of the independent variables were assessed using self-reported information from LST instructors and local coordinators. Not all teachers completed surveys, and it is possible that teachers who were less supportive of the program did not participate in the assessment. In addition, data were cross-sectional, and causal inferences regarding predictors and outcomes could not be made. These limitations emphasize the need for additional studies assessing predictors of implementation quality in community-based replications of prevention programs. More research is needed to identify the degree to which communities can replicate programs successfully, and more quantitative analyses of factors that influence the successful adoption of new programs are needed. In particular, studies utilizing multiple respondents, longitudinal designs, and random assignment are needed.

Despite the levels of program monitoring and support provided in the Blueprints Initiative, it is telling that schools nonetheless faced many challenges during implementation. Interviews with program staff indicated barriers related to finding room in the school schedule for the program, with schools trying to avoid taking time away from academic subjects but also risking student resentment if electives or free time was used to teach LST. Most schools struggled to some extent with gaining full support from key participants, including site coordinators, instructors, principals and other school administrators. Finally, student misbehavior and classroom management difficulties were reported by many teachers, particularly when trying to implement the interactive components in the curriculum.

In most cases, schools were able to overcome barriers, at least over the course of the three-year project. Schools also varied in their willingness to identify challenges to research staff. Most schools seemed reluctant to ask for TA, even though they were strongly encouraged at the start of the project and in annual reports to utilize the free technical assistance available. In many cases, Blueprints staff did not learn about problems until site visits were made, and requests for visits by LST trainers were rare. We quickly learned that "no news was good news" did not apply to the schools participating in this project and that *proactive *TA (versus waiting for schools to contact TA providers) was needed to identify and solve implementation challenges.

During the past decade, we have learned much about the importance of implementation fidelity in achieving effective outcomes, and about what is required to attain a high-quality implementation. The primary lesson learned in this project was that schools can effectively replicate evidence-based drug prevention curricula, including teaching the majority of required lessons, content, and activities in a manner that engages students. Doing so involves much planning and problem-solving, and schools must be ready to encounter challenges. Even when problems are encountered and solved, they may reappear later in implementation and have to be resolved again. The provision of technical assistance and implementation monitoring is critical for identifying and overcoming barriers to implementation. Implementation fidelity was especially high in this project due to the level of TA support provided by NHPA and the implementation monitoring by Blueprints staff. Under natural conditions, schools may not achieve the same level of fidelity as found in this project, but even without this high level of support schools can still improve implementation fidelity (and hence outcomes). Purchasing a curriculum and asking teachers to implement, in the absence of training, support, and monitoring, will seldom be sufficient. School administrators must be proactive in supporting teachers' efforts to replicate programs. This includes choosing programs carefully to ensure fit, ensuring that teachers are trained in the program model and in classroom management skills, building an internal mechanism of ongoing support (e.g., assigning a trained program coordinator), and instituting at least some minimal form of internal monitoring that provides corrective feedback to teachers and administrators regarding implementation.

## Competing interests

The author(s) declare that they have no competing interests.

## Authors' contributions

The replication project was directed by SFM, and AF and SA were project managers at different time intervals throughout the project, overseeing field staff and data collection. SFM conceived of the study, and SFM and AF participated in the study design, statistical analyses, and writing. AF had a major role in preparing the first draft. SA prepared the file for analysis. All authors read and approved the final version.

## Supplementary Material

Additional file 1 LifeSkills Training Implementation Fidelity – Site Selection and Training. Detailed information is provided on site selection and training during the course of the grant. Click here for file
