# Supplementary material for: Implementing the LifeSkills Training drug prevention program: factors related to implementation fidelity
Source: Implement Sci. 2008 Jan 18;3:5. doi: 10.1186/1748-5908-3-5 (PMC2265741; doi:10.1186/1748-5908-3-5)
Supplement: Additional file 1 — LifeSkills Training Implementation Fidelity – Site Selection and Training. Detailed information is provided on site selection and training during the course of the grant. [file 1748-5908-3-5-S1.doc]

**LifeSkills Training Implementation Fidelity - Site Selection and Training**

**Site selection**

The site selection process involved an application review and a feasibility site visit. The term “site” refers to the entity applying. A site application could involve one or more schools within a district or one or more school districts within a locality. In a few instances, the locality spanned multiple counties or parishes. In 14 sites, an outside prevention agency was the applicant. These agencies partnered with schools to provide trained instructors to teach LifeSkills Training (LST), relieving teachers of that task. Table 1 shows site locations and number of schools.

There were three separate announcements by the Office of Juvenile Justice and Delinquency Prevention (OJJDP) in three subsequent years to provide training and technical assistance to sites that wanted to adopt the LST program. Only Grant 1 sites responded to the Request for Proposal (RFP) by writing a 30 page grant application. Grant 2 and 3 sites applied directly to the Center for the Study and Prevention of Violence (CSPV) by completing an application designed by CSPV. This change in the application process enabled CSPV to collect more pertinent information on a site’s commitment and plan for implementation very early in the grant review process. The application was designed to encourage sites to think carefully about logistical details involved in implementation (e.g., when the program would be taught, in what class and by whom) in hopes that potential problems would surface earlier than later, providing a better chance for resolution. If potential problems were discovered in the application stage, a call was made to the site to try to resolve the issue. For instance, a site might have stated on the application that they would implement LST in PE, with a mixture of sixth to eighth grade students. Since level 1 of LST is intended to be taught to only one grade (sixth or seventh), we would have considered this a breach of fidelity and asked the site to reconsider the placement of the program or find a way to separate the different grades. This helped sites to tackle difficult issues early.

If potential problems discovered during the application stage were deemed to be resolvable, a feasibility visit was scheduled. A CSPV representative and a LST-certified national trainer under contract with National Health Promotion Associates, Inc. (NHPA), the dissemination agency for LST, met with key stakeholders at the site. Attendance by the site coordinator, principal, and at least one teacher representative from every school within the site was required. These visits generally lasted from three to four hours, and included a presentation by the LST trainer that covered the core components of the program, followed by a question and answer period. The CSPV representative followed this segment with an explanation of the requirements of the grant and a semi-structured interview with all participants to obtain information on the overall level of commitment, the site’s plan for implementation, and their willingness to conduct the program with fidelity and allow teachers to be observed while implementing LST lessons. After this visit, with input from the NHPA certified trainer, a decision was made by CSPV as to whether or not to accept the site. In some instances, it was clear that some of the schools within a site were committed to the project and willing to implement the program with fidelity, while others were not. Thus, there were instances where schools within a site were asked to withdraw prior to site selection.

The number of sites that applied and were selected follows:

**Grant one**

In December, 1998, OJJDP issued an RFP for communities that wanted to address drug prevention in their schools/communities by implementing the LST program. Twenty-seven sites responded to the RFP, and from these, 14 sites were approved to receive the technical assistance and curriculum materials offered by this grant, which would be provided through CSPV and NHPA. No monetary awards were made to the sites. The 14 sites included 78 schools and approximately 39,214 students who were projected to receive LST over the three-year period of the grant.

On June 25, 1999, a second RFP was issued by OJJDP for implementing LST. Thirty sites applied for this grant, and five were initially rejected. Among the 25 remaining sites, 21 passed through all steps of the feasibility process. The 21 sites included 64 schools and approximately 37,450 students who were projected to receive LST over the three-year period.

Combined, 57 sites responded to the first and second RFPs. Of these, 35 were selected to receive the training and technical assistance provided by the grant. The number of participating schools changed each year, as some schools withdrew from the grant and some schools were added because of school restructuring or high schools (grade nine) that were added in the third year of implementation. By the end of year three, these sites included 156 schools in 33 sites and approximately 74,073 students. There were two complete site failures, representing three schools, and an additional nine schools across six sites that withdrew their participation from the project (see Addendum Table 2). Three of these schools withdrew prior to year one implementation.

**Grant two**

On March 1, 2000, OJJDP issued an announcement of a funding opportunity for communities that wanted to address drug prevention in their schools/communities by implementing the LST program. This time, sites applied for the program using an application process developed by CSPV.. Forty-six sites responded to the announcement, and from these, 35 sites were approved, after feasibility visits, to receive the technical assistance and curriculum materials offered through the grant. By the end of year three, there were 130 schools in 33 sites that completed implementation, and approximately 50,154 students who received the curriculum. There were two complete site failures, representing two schools, and an additional seven schools across six sites that withdrew their participation from the project. Three of these schools withdrew prior to year one implementation.

**Grant three**

On March 14, 2001, OJJDP issued the last announcement to fund new LST sites. Sites could apply directly to CSPV. Fifty-three sites responded to the announcement, and from these, 38 feasibility visits were conducted, and ultimately 35 sites were approved to receive the technical assistance and curriculum materials offered through this grant. Four of the original applying sites were combined into two sites because of close proximity to one another. By the end of year three, there were 117 schools in 33 sites that completed implementation, and approximately 48,128 students who received the curriculum. There were two complete site failures, representing two schools, and an additional six schools across five sites that withdrew their participation from the project. Three of these schools withdrew prior to year one implementation.

**Training**

The grant provided for one training per site for each year of the grant, which consisted of a two-day initial training in year one, followed by booster sessions in the subsequent years. Booster trainings were one or two days (both models have proven effective through research), depending on the needs of the site (number and level of training teachers needed), although each site was encouraged to conduct two-day booster workshops. Trainings were coordinated by NHPA, who began the planning process several months prior to the actual training date, typically during the spring in anticipation of an early fall training. The sites were required to address all logistical concerns, including submission of the number of participants attending, while NHPA ordered all materials and arranged travel for their trainers. Once all three grants were operating at the same time, and most of the sites requested their training within the same time frame, NHPA utilized multiple staff to cover the demands of the planning process. They also employed numerous certified LST trainers who were located around the country to accommodate all training needs.

Training objectives included a review of the background theory, research and rationale behind the LST program, familiarizing participants with the curriculum, developing the teaching skills necessary to successfully implement the program, discussion of implementation issues, and an opportunity for participants to practice teaching selected portions of the LST curriculum. Trainers utilized the various teaching skills (didactic, discussion, demonstration, and behavioral rehearsal) during the training in order to reinforce these methods of program delivery. CSPV required that all program facilitators and site coordinators attend the initial training in the first year. CSPV made a point to emphasize the importance of the coordinators attending training, in order to become more knowledgeable about the program and the necessary requirements for proper implementation, as well as to show support to implementing staff. Data were not collected on exactly how many site coordinators attended the trainings in their entirety, although all coordinators attended at least part of the training. If coordinators were replaced at any point during implementation, the new coordinators were also asked to attend the upcoming site training. Classroom observers were also required and paid to participate in the training to familiarize themselves with the lesson objectives. School administrators and other support staff (i.e., counselors) were highly encouraged to attend, but in many instances, administrators were unable to reserve the time away from their duties to participate. Many, however, made a point to briefly attend for introductions and to express their support to the teachers.

Over the course of the grant, a total of 312 on-site trainings were conducted, and included 4,369 participants. Teachers who taught multiple levels of the curriculum often attended training each year. At the completion of training, participants completed a survey. Over 80% (3,529) of participant surveys were collected. They were asked to rate the overall quality and organization of the training workshop, and indicate their level of confidence in properly implementing the program as a result of training. Participants also rated aspects of the trainer(s), including poise, knowledge of the program, rapport and communicability, level of enthusiasm, ability to answer questions/concerns, and an overall trainer rating. They could also comment about what they felt were the most and least valuable aspects of the training, the length of the training, if they felt any areas were not adequately covered, if they felt a need for additional training, and any other comments. Collected data were fed back to the individual sites in their year-end reports. The reports were also disseminated to NHPA, so that trainers could learn how to better prepare for upcoming trainings with their sites. In general, participant sentiment regarding the trainings was very positive and sites were very satisfied with the trainings and their trainers. They liked learning about the program, and having opportunities to practice the lessons in front of their peers for feedback, as well as allowing time to discuss any implementation issues or concerns they might have. If any problems were indicated, CSPV worked with NHPA and the site to meet the needs of the site, which sometimes meant using a different trainer in subsequent years.

It was inevitable that the project experienced teacher turnover. This occurred for several reasons, including moving the program to a new subject area, new school hires, replacements for sick teachers, etc. Every effort was made to accommodate sites that needed to train additional teachers after their official site training had been completed. Most often, if several new teachers needed to be trained, an additional training was provided to the site. NHPA required at least six participants in order to classify the workshop as a training (for billing purposes). If only a few teachers needed training, a technical assistance visit was arranged to complete training. Twenty-eight such TA visits were completed over the course of the project. The final option was to send teachers to a nearby training at another site, if possible. There were, however, instances in which the site did not inform either CSPV or NHPA of new staff until implementation had ended. In these few instances, the students were either taught by an untrained teacher or did not receive the program. The data collected does not indicate how many students this affected, although it only occurred at ten sites.

**Technical assistance**

Grant funding enabled NHPA representatives to provide technical assistance (TA) to sites in each year of the project, in the form of telephone and e-mail consultation, and on-site visits. A total of only 67 on-site TA visits were conducted throughout the course of the grant. As mentioned above, 28 of these were to conduct training for additional teachers. The remainder of the visits were conducted at various times for each site. Those sites that requested a visit prior to implementation discussed potential upcoming issues or concerns with their NHPA representative in order to avoid issues during program implementation. Other sites requested a visit during their implementation, in order to obtain clarification on issues or to review the progress of implementation. The remainder of visits occurred once implementation was complete, in order for teachers to provide feedback to each other and to prepare for the next year’s implementation. Reports to CSPV varied on why many sites did not take advantage of this service. Many of the NHPA representatives only corresponded with their sites if they were contacted. CSPV field representatives discovered on many of their site visits that teachers were either unaware or they had forgotten that TA was available to them. Sites were reminded each year that TA was available and encouraged to contact their TA provider with any issues or concerns. Finding time during the school year to schedule a visit was often difficult, and in these cases, TA was just not sought, or the sites corresponded with the TA provider via telephone or e-mail. Most teachers reported that they did not feel the need for additional assistance, particularly after their initial year of implementing the program and they were more comfortable delivering the curriculum.

**Table 1. LST sites and number of schools participating at the end of the grant (does not include schools that withdrew that are listed in Addendum Table 2)**

| **Grant 1** |  | **Grant 2** |  | **Grant 3** |  |
| --- | --- | --- | --- | --- | --- |
| **Site Name** | **# Schools** | **Site Name** | **# Schools** | **Site Name** | **# Schools** |
| Alexandria, LA | 21 | Amarillo, TX | 3 | Bartlesville, OK | 1 |
| Brooklyn, NY | 2 | Baton Rouge, LA | 2 | Boulder/Westminster, CO | 4 |
| Chicago, IL | 1 | Benecia, CA | 1 | Caldwell, ID | 1 |
| Cortez, CO | 1 | Bryan, TX | 3 | Cedar Rapids, IA | 1 |
| Des Moines, IA* | 2 | Buffalo, WY | 2 | Clarksdale, MS* | 12 |
| E. Cleveland, OH | 6 | Cache Co., UT | 3 | Cleveland, OH* | 2 |
| Elizabeth, NJ | 4 | Carbon Co., WY | 2 | Dallas, TX* | 1 |
| Fontana, CA | 7 | DeKalb Co., AL | 6 | Decatur, AL | 0 |
| Foxborough, MA | 2 | Edmonds, WA | 2 | Durham, NC | 4 |
| Hiram, ME | 6 | Elizabeth, NJ | 5 | Evanston, WY* | 1 |
| Houston, TX | 1 | Evansville, IN | 12 | Evansville, IN | 24 |
| Hudson, NY | 1 | Greece, NY | 4 | Fitzgerald, GA | 0 |
| Johnstown, PA | 1 | Hamilton, MT | 2 | Florence, NJ | 1 |
| Juneau, AK* | 0 | Hartford, CT | 3 | Granite Falls, WA | 2 |
| Kansas City, MO | 2 | Hays, KS | 2 | Hettinger, ND | 1 |
| Kirksville, MO* | 0 | Hemet, CA | 7 | Humbolt, IA | 1 |
| Little Rock, AR | 8 | Lincoln Co., NM | 3 | League City, TX | 9 |
| Livonia, NY | 2 | Louisville, CO | 0 | Lynwood, WA | 2 |
| Logan Co., OH | 4 | Lynn, MA | 4 | Marblehead, MA | 1 |
| Lubec, ME | 12 | Madison, FL | 2 | Metuchen, NJ | 1 |
| Madison, WI | 3 | Madison, WI | 2 | Monroe, WA | 4 |
| Marksville, LA | 3 | Mahnomen, MN | 3 | New Brighton, PA | 1 |
| Mason City, IA | 2 | Marion Co., KS | 7 | Niagara Falls, NY | 3 |
| Moses Lake, WA | 2 | Minneapolis, MN | 4 | Plentywood, MT* | 4 |
| Oakhurst, NJ | 2 | Natchitoches, LA | 10 | San Jose, CA | 6 |
| Plaistow, NH | 1 | Newton, IA* | 2 | San Pablo, CA | 1 |
| Rochester, NY* | 23 | Norwich, CT | 2 | Spokane, WA* | 4 |
| Salt Lake City, UT* | 15 | Norwood, CO | 1 | Springfield, MO | 5 |
| Springfield, MA | 7 | Ontario Co., NY | 8 | Utica, NY | 2 |
| Stover, MO | 1 | Portage, WI | 2 | Van Horn, TX | 1 |
| Utica, NY* | 2 | Prineville, OR | 1 | Washington, DC | 4 |
| Vallejo, CA | 4 | Sabine, LA | 7 | Waushara Co., WI | 2 |
| Vero Beach, FL | 4 | Shreveport, LA | 0 | Wayne Co., NY | 5 |
| W. Palm Beach, FL | 2 | Sweet Home, OR | 6 | Wenatchee, WA | 3 |
| Willingboro, NJ | 2 | Wenatchee, WA | 7 | Winter Haven, FL* | 3 |
|  | 156 |  | 130 |  | 117 |

* Program was taught by staff from an outside agency at some or all schools.

**Addendum Table 2. Site and School Failures**

| **Grant 1** |  | **Grant 2** |  | **Grant 3** |  |
| --- | --- | --- | --- | --- | --- |
| **Site Name** | **# Schools** | **Site Name** | **# Schools** | **Site Name** | **# Schools** |
| ***Full Site Failures*** |  | ***Full Site Failures*** |  | ***Full Site Failures*** |  |
| Juneau, AK | 2 | Louisville, CO | 1 | Decatur, Al* | 1 |
| Kirksville, MO | 1 | Shreveport, LA | 1 | Fitzgerald, GA | 1 |
| ***School Failures*** |  | ***School Failures*** |  | ***School Failures*** |  |
| Alexandria, LA | 2 | DeKalb Co., AL | 1 | Durham, NC* | 1 |
| Brooklyn, NY | 1 | Hamilton, MT* | 1 | Evanston, WY* | 1 |
| Des Moines, IA* | 1 | Lincoln Co., NM | 1 | Spokane, WA | 1 |
| Lubec, ME | 3 | Minneapolis, MN* | 1 | Washington D.C. | 2 |
| Oakhurst, NJ* | 1 | Ontario Co., NY | 2 | Boulder/Westminster, CO* | 1 |
| Vallejo, CA* | 1 | Webster Parish*^1^ | 1 |  |  |
|  | 12 |  | 9 |  | 8 |

*Schools that withdrew from the project prior to year one training and implementation.

^1^Webster Parish applied with the Nachitoches/Sabine Parishes.
